# Bioaugmentation by *Pediococcus acidilactici* AAF1-5 Improves the Bacterial Activity and Diversity of Cereal Vinegar Under Solid-State Fermentation

**DOI:** 10.3389/fmicb.2020.603721

**Published:** 2021-01-28

**Authors:** Qiang Zhang, Cuimei Zhao, Xiaobin Wang, Xiaowei Li, Yu Zheng, Jia Song, Menglei Xia, Rongzhan Zhang, Min Wang

**Affiliations:** ^1^State Key Laboratory of Food Nutrition and Safety, Key Laboratory of Industrial Fermentation Microbiology, College of Biotechnology, Ministry of Education, Tianjin University of Science and Technology, Tianjin, China; ^2^Tianjin Tianli Duliu Mature Vinegar Co., Ltd., Tianjin, China

**Keywords:** solid-state fermentation vinegar, Tianjin Duliu mature vinegar, canonical correspondence analysis, bioaugmentation, *Pediococcus acidilactici*

## Abstract

Bioaugmentation technology may be an effective strategy to improve the solid-state fermentation rate and utilization of raw materials for traditional vinegar production. The relationship between bacteria and fermentation process was analyzed to rationally design and perform bioaugmented solid-state fermentation of the Tianjin Duliu mature vinegar (TDMV). Fermentation process was highly correlated with *Acetobacter, Lactobacillus*, and *Pediococcus* contents, which were the core functional microorganisms in TDMV fermentation. *Pediococcus acidilactici* AAF1-5 was selected from 20 strains to fortify the fermentation due to its acidity and thermal tolerance. Bioaugmentation was performed in the upper layer of TDMV fermentation. *P. acidilactici* AAF1-5 colonized and then spread into the lower layer to improve the fermentation. Result showed that the fermentation period was 5 days less than that of the control. Meanwhile, the non-volatile acid, lactic acid, amino nitrogen, and reducing sugar contents in the bioaugmented TDMV increased by 53%, 14%, 32%, and 36%, respectively, compared with those in the control. Bioaugmentation with *P. acidilactici* AAF1-5 not only improved the utilization of starch from 79% to 83% but also increased the bacterial community diversity.

## Introduction

**FIGURE 1:**
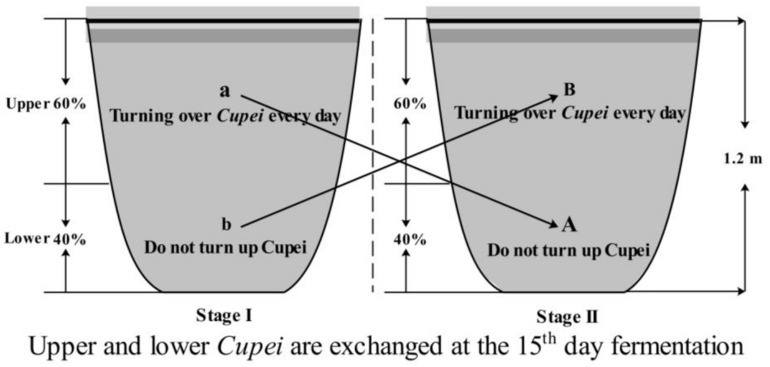
Flow chart of TDMV solid-state acetic acid fermentation.

Tianjin Duliu mature vinegar (TDMV) is one of the famous Chinese vinegars produced using traditional solid-state fermentation technology, with sorghum, millet, bran, and rice husk as raw materials. *Daqu* is added to start TDMV saccharification and fermentation, and then alcoholic fermentation and acetic acid fermentation (AAF) are successively carried out (Nie et al., 2013). Different from that of other vinegars, the AAF of TDMV is divided into two stages, which is the most important process for flavor compounds formation. As described in Figure 1, during the first 15 days (stage I), only the upper layer of the vinegar mash (called *Cupei*) “a” was turned over every day. On day 15, the upper and lower layers of *Cupei* are exchanged and fermented for another 15 days (stage II), with turning over of *Cupei* “B” every day.

In TDMV fermentation, the raw materials are decomposed and metabolized into sugars, organic acids, amino acids, and multiple-flavor substances under the action of microorganisms (Hutchinson et al., 2019). Various types of microorganisms that derive from starter cultures or are naturally enriched from the environment, are involved. The cooperation of various microorganisms produces the unique flavor of vinegar (Ho et al., 2017). Fungal contents (mainly mold and yeast) decrease intensively during the AAF of TDMV, and bacteria become the predominant microorganisms (Nie et al., 2013). Acetic acid bacteria (AAB) and lactic acid bacteria (LAB) are the most abundant and important functional microorganisms (Li, 2019). The concentration of AAB increases during fermentation, whereas that of LAB decreases (Zheng et al., 2018). In AAF of Shanxi aged vinegar, the dominant genera are *Lactobacillus* and *Acetobacter*, and *L. helveticus* and *A. pasteurianus* are predominant species, accounting for more than 70% of the total bacteria concentration (Zhang et al., 2020). In AAF of Zhenjiang aromatic vinegar, *Lactobacillus* is predominant in the early stage of AAF (days 0–9), while *Acetobacter*, *Lactococcus*, *Gluconacetobacter*, *Enterococcus*, and *Bacillus* are prevailing in the later stage of AAF (days 10–18) (Wang et al., 2016).

The fermentation process is closely related to the structure of microbiota (Hamdouche et al., 2019). Multivariate statistical analyses, such as canonical correspondence analysis (CCA), could determine the relationship between the microbiota and physicochemical indices (Nie et al., 2017). A high correlation exists between some microorganisms and some flavor compounds (Ai et al., 2019). However, some environmental factors, such as acidity, pH, and reducing sugar, could influence the growth and metabolism of microorganisms (Zheng et al., 2018). The insufficiency of functional microorganisms could also result in prolonged production period, low utilization of raw materials, and even unstable quality of products (Solieri and Giudici, 2009; Song et al., 2019; Tang et al., 2019). Bioaugmentation could be an effective technology to solve these problems. Bioaugmentation is a method that uses specific functional strains or microbial combinations for biological enhancement to achieve the purpose of improving fermentation efficiency or directional change of flavor components (Yang et al., 2016a,b). The bioaugmentation in the fermentation of semi-hard cheese by adding *Micrococcus* sp. INIA 52 shortened the maturation period from 18 days to approximately 14 days (Morales et al., 2005). The bioaugmentation of *Bacillus velezensis* and *Bacillus subtilis* in traditional *Daqu* increased the abundance of *Bacillus*, *Lactobacillus*, and *Candida*, and improved the liquefying, saccharifying, and esterifying powers of *Daqu* (He et al., 2019). The bioaugmentation of *Monascus purpureus* in Sichuan bran vinegar inhibited several pathogen-associated microorganisms and increased the abundance of *Lactobacillus* and *Acetobacter* by 24.65% and organic acids, aromatic esters, and alcohols by 1.95, 2.30, and 3.55 times, respectively (Ai et al., 2019).

However, which kind of microorganism should be enhanced must be rationally analyzed and designed before applying bioaugmentation. In this study, the correlation of microorganisms with the fermentation process and the internal environment of TMVD were analyzed. Then, TMVD fermentation was bioaugmented using a strain of LAB isolated from the indigenous source of TMVD in accordance with the relationship between the bacteria and the fermentation process. In particular, the effect of bioaugmentation was revealed by comparing the microbiota compositions.

## Materials and Methods

### Experimental Materials

*Cupei* “a–A” and “b–B” were sampled at 0, 5, 10, 15, 20, 25, and 30 days during TDMV fermentation. A five-point sampling method was used to collect samples from different positions. These samples were placed in sterile bags in an ice box. They were collected in three parallels, and each sample weighed approximately 200 g. The first 0–15 days of AAF was defined as stage I, where 0–5, 5–10, and 10–15 days were presented as early stage I, middle stage I, and late stage I, respectively. The other 15 days of AAF was defined as stage II, where 15–20, 20–25, and 25–30 days were referred to as early stage II, middle stage II, and late stage II, respectively.

### Strains

All LAB strains were isolated from the *Cupei* of TDMV. The strain of *Pediococcus acidilactici* AAF1-5 (CGMCC 12062) deposited in the China General Microbiological Culture Collection Center was selected for the bioaugmentation of TDMV fermentation.

### Analysis of the Physicochemical Properties of the Fermentation Process

Five grams of the *Cupei* sample was added into 45 mL of deionized water, shaken at 180 r/min for 1 h, and centrifuged for 10 min at 15,000 × g to obtain the supernatant. The total acid, amino nitrogen, and reducing sugar contents were determined following the previous methods (Zheng et al., 2018). An Agilent 1260 high-performance liquid chromatography system (Agilent Corp., Karlsruhe, Germany) was employed for organic acid analysis with Aminex HPX-87H ion exclusion column (7.8 × 300 mm; i.d., 5 μm). Organic acids were detected at 215 nm. The mobile phase consisted of H_2_SO_4_ (5 mmol/L) with a flow rate of 0.6 mL/min. The column temperature was 30°C. The organic acids were quantified using external calibration curves (Nie et al., 2013).

### Microbial Community Analysis

Metagenomic samples were prepared on the basis of previous methods (Zheng et al., 2018). Primers 338f and 518r were used to amplify the V3 − V4 region of the bacterial 16S rDNA (Muyzer et al., 1993; Nie et al., 2013). Then, PCR fragments were sent to GENEWIZ, Inc. (Suzhou, China) for Illumina high-throughput sequencing. The sequencing results were compared via BLAST on Genbank^1^. These sequence data were deposited in the NCBI Sequence Read Archive (SRA)^2^ with accession number SRR11951261. The genus or species with the highest identity was selected as the identification result.

α-Diversity indices (including ACE, Chao1, Simpson, and Shannon indices) were quantified in terms of operational taxonomic unit (OTU) richness to indicate the microbial diversity (Nie et al., 2013).

### Bioaugmentation Fermentation of TDMV

*P. acidilactici* AAF1-5 was statically cultured in MRS medium at 37°C for 24 h. Then, 500 mL of broth was centrifuged at 8,000 × g for 10 min and washed with sterilized physiological saline. Subsequently, the precipitated bacteria were resuspended in 100 g/L of lactose solution. The bacterial suspension was pre-frozen in the refrigerator at 4°C for 1 h and then at −80°C for 24 h. It was placed in a lyophilizer under the cold trap temperature of −50°C and pressure of 1.0 Pa and then lyophilized for 48 h to obtain the strain agent.

The fermentations were performed in a ceramic pot (Figure 1). The strain agent was used for the bioaugmentation of TDMV fermentation (bioaugmentation group, BG). The specific experimental program was as follows: 10 g of the strain agent (5.66 × 10^10^ CFU/g) was dissolved in 100 mL of sterilized water and then added into *Cupei* “a” on day 1 of AAF. For the control group (CG), 100 mL of lactose solution (100 g/L) was added. Each fermenter consisted of approximately 100 kg of *Cupei*. The sampling position of *Cupei* “a” and “B” was 30 cm from the surface, while that of *Cupei* “b” and “A” was 90 cm from the surface.

### Determination of the Utilization Ratio of Starch Raw Materials

Ten grams of *Cupei* was placed in 250 mL flask and added with 100 mL of 10% diluted hydrochloric acid solution. The reaction was conducted in water bath at 80-90°C for 1 h. Then, the solution was cooled to room temperature and adjusted to pH 7.0 with saturated sodium hydroxide solution. The solution was filtered and volumized into 500 mL. The reducing sugar content was measured in accordance with the previous method (Zheng et al., 2018). Starch utilization was calculated using Formulation 1.

(1) $$\begin{array}{c} \text{Starch}\text{utilization}=\\ \frac{\text{total}\text{acid}\text{in}Cupei+\text{alcohol}\text{conten}\text{in}Cupei\times 1.304}{\left(\text{total}\text{raw}\mathrm{starch}-\mathrm{total}\text{sugar}\text{in}Cupei\times 0.9\right)*0.740} \end{array}$$

### Statistical Analysis

All experiments were performed in triplicate. The results were expressed as mean values with standard error. *T*-test was used for significance analysis on the IBM SPSS Statistics 22.

Canoco software version 5.0 for Windows was used to reveal the correspondence between the bacterial community and the physicochemical indicators.

Principal component analysis (PCA) and intergroup difference analysis were used to evaluate the bacterial community differences among samples on the Canoco software for Windows 5.0 and Minitab 17 statistical software, respectively.

## Results

### Correspondence Between Bacterial Community and Fermentation Process

**FIGURE 2:**
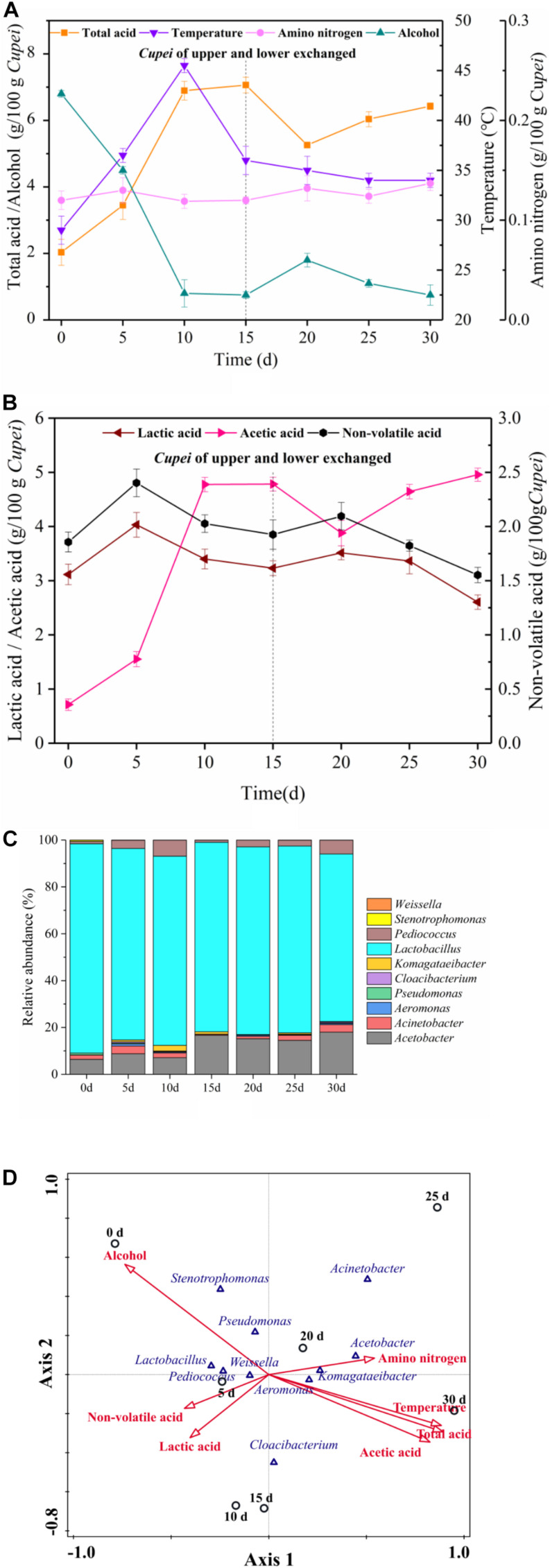
Time curves and correspondence between microorganisms and fermentation process. On day 15, the upper and lower layers of *Cupei* are exchanged. **(A)** Physicochemical indexes, **(B)** organic acids, **(C)** bacterial community, and **(D)** correspondence between bacteria and fermentation process.

The changes in the main physicochemical properties of *Cupei* “a–A” and the construction of bacterial community during the AAF of TDMV are shown in Figure 2. As shown in Figures 2A,B, the total acid increased in stages I and II of AAF. The change in alcohol content was opposite to that in the total acid content. Lactic acid and acetic acid are the most important organic acids in vinegar, accounting for more than 90% of the total organic acid (Verzelloni et al., 2007). In the present study, the acetic acid content rapidly increased in stage I and slowly increased in stage II, whereas the lactic acid content slightly increased and then decreased in both stages. The amino nitrogen content increased slightly due to the continuous hydrolysis of the crude protein in the raw materials under the action of microorganisms during fermentation. The temperature increased in the early and middle stage I and then decreased in the late stage I. However, the temperature slowly decreased in stage II.

The bacterial community was also analyzed, and a total of 348 MB of data were produced in the Illumina MiSeq high-throughput sequencing platform, which included 4,34,548 reads. Chimera-free sequences were clustered in OTU defined by 97% similarity. Representative sequences were classified in accordance with previously described methods (Kostic et al., 2012). A total of 138 OTUs (relative abundance/OTU of more than 0.5%) were generated from seven samples belonging to 10 genera, as shown in Figure 2C. The relative abundance of *Lactobacillus* genus was the highest, accounting for more than 75% of the total bacteria. Two AAB genera, namely *Acetobacter* and *Komagataeibacter*, were detected. The relative abundance of *Acetobacter* increased from 6.38% in early stage I to 18.18% in late stage II. The relative abundance of *Pediococcus* in early stage I was 1.02%, which increased to 5.14% in late stage I. In stage II, its abundance increased from 1.18% to 5.91%. *Aeromonas*, *Acinetobacter*, and *Pseudomonas* were also detected. The relative abundance of *Acinetobacter* gradually increased in both stages of AAF. However, the role of these microorganisms in vinegar fermentation is still unclear.

The growth and metabolism of microorganisms play a vital role for the fermentation process. Meanwhile, environmental factors affect the growth and metabolism of microorganisms. In this study, CCA was used to analyze the correlation between the bacterial community and the main physicochemical indices during the AAF of TDMV. As shown in Figure 2D, non-volatile acid and lactic acid were closely related to the stage I samples, indicating their accumulation in this stage. The LAB mostly associated with non-volatile acids and lactic acid were *Lactobacillus*, *Pediococcus*, and *Weissella*. With the increase in the acidity of *Cupei*, the relative abundance of LAB and the lactic acid content decreased. Total acid and acetic acid were closely related to the stage II samples, indicating that they were mainly accumulated in this stage. The AAB mostly associated with total acid and acetic acid were *Acetobacter* and *Komagataeibacter*. Alcohol showed a negative correlation with *Acetobacter* and *Komagataeibacter*, mainly because it was rapidly oxidized to acetic acid by these AAB during fermentation. Amino nitrogen demonstrated a positive correlation with stage II, because the increase in the acidity of *Cupei* is favorable to the decomposition of macromolecular proteins and peptides (Duong et al., 2019; Guo et al., 2019). The small angle between temperature and acetic acid indicated their high correlation. The metabolism of microorganisms produced a large amount of biological heat, resulting in increased *Cupei* temperature (Xia et al., 2019). The high correlation between temperature and acetic acid suggested that the increase in temperature was mainly generated by alcohol oxidation. Temperature and lactic acid were projected in different directions, indicating that temperature possibly inhibited the formation of lactic acid. These results showed that *Acetobacter, Lactobacillus*, and *Pediococcus* were the core functional microorganisms in the AAF of TDMV. Among them, *Acetobacter* showed an important effect on the conversion of alcohol, while *Lactobacillus* and *Pediococcus* affected the formation of lactic acid.

### Bioaugmentation Fermentation of TDMV

**FIGURE 3:**
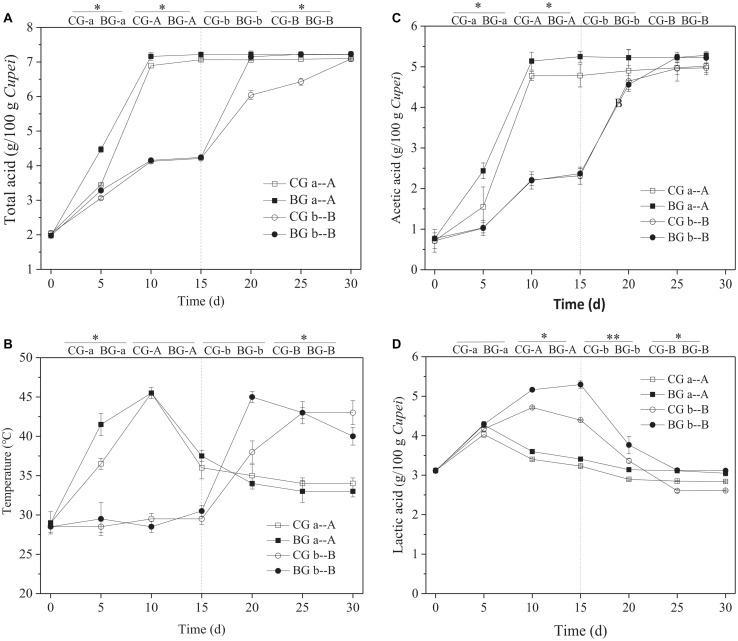
Time curves of the fermentation process. **(A)** Total acid, **(B)** temperature, **(C)** acetic acid, and **(D)** lactic acid. On day 15, the upper and lower layers of *Cupei* are exchanged. **Indicates there is a significant difference at *P* < 0.01 level, *indicates there is a significant difference at *P* < 0.05 level.

High temperature and highly acidic environment inhibit the growth and metabolism of microorganisms (Xiao et al., 2017). Therefore, the fermentation characteristics and tolerance of 20 strains of LAB (strain nos. from AAF1-1 to AAF1-20) isolated from TDMV *Cupei* were analyzed. The strain of *P. acidilactici* AAF1-5 was selected for the bioaugmentation of TDMV in accordance with the tolerance against thermal and acid stresses (Supplementary Figure 1). As shown in Figure 3A, the total acid contents in *Cupei* “a–A” did not change after 10 days of AAF. However, the acid production rate was higher in the early stage I of the BG than in the CG. The total acids in the BG and CG of *Cupei* “b–B” were the same in stage I, because bioaugmentation was only performed in *Cupei* “a–A.” In addition, 60% of *Cupei* was turned over every day; thus, the strain of *P. acidilactici* AAF1-5 that was added into the *Cupei* “a” of the BG gradually spread into *Cupei* “b” due to mass transfer. This special operation of TDMV determined the right site and timing to perform bioaugmentation in *Cupei* “a” at the beginning of AAF. After exchanging the upper and lower layers of *Cupei* on day 15 of fermentation, the acid production rate of the BG in *Cupei* “B” became higher than that of the CG. The termination of the increase in total acid is usually the index for the end of AAF. At 24 d, the contents acetic acid and lactic acid did not change in the BG, and total acid content reached the maximum, which was 5 days less than that in the CG.

The AAF of TDMV was performed in a ceramic pot without a temperature control system. The change in temperature is almost due to the metabolism of microorganisms. Thus, temperature is an important parameter that indicates the metabolic activity of microorganisms (Zheng et al., 2018). The *Cupei* “a” temperature of the BG increased more quickly from 0 to 5 days (2.5°C/d) than that of the CG (1.5°C/d), indicating the more active metabolism of microorganisms (Figure 3B). The *Cupei* “b” temperatures of the BG and the CG were the same in stage I, because of the same metabolic activity of microorganisms. After the upper and lower layers of *Cupei* were exchanged (15–25 d), the *Cupei* “B” temperature of the BG was higher than that of CG. The increase in temperature was correlated with the production rate of total acid. This result demonstrated that the metabolism in the upper layer of *Cupei* (“a” and “B”) was more active than that in the lower layer (“b” and “A”) due to the sufficient mass transfer (e.g., oxygen and water) resulting from the turning over *Cupei*.

As shown in Figures 3C,D, acetic acid was mainly produced in the upper layer of *Cupei* (“a” and “B”), whereas lactic acid was produced in the lower layer of *Cupei* (“b” and “A”). The acetic acid content in *Cupei* “a” did not increase after it was exchanged into the lower layer of the fermenter, whereas that in *Cupei* “B” rapidly increased after 15 d. At the end of AAF, the acetic acid in *Cupei* “B” reached the same content as that in *Cupei* “A,” indicating that almost all of the alcohol was oxidized. The *Cupei* “a” of the BG showed a faster acetic acid production rate (1.05 g/g *Cupei*/d) than that of the CG (0.96 g/g *Cupei*/d), and the acetic acid contents in *Cupei* “A” and *Cupei* “B” of the BG were higher than those of the CG even though no AAB were added into the BG. The lactic acid content in *Cupei* “a–A” increased in the early stage I and then decreased. However, the lactic acid contents in *Cupei* “a–A” and “b–B” of the BG were higher than those of the CG due to the bioaugmentation of *P. acidilactici* AAF1-5. These results indicated that *P. acidilactici* AAF1-5 could colonize and play a role in the AAF of TDMV due to its acidification capacity and thermal tolerance.

After AAF, 250 L of hot water (approximately 90°C) was added into each fermenter to soak the *Cupei*. Then, the leached vinegars were compared. As shown in Table 1, no significant difference in the total and acetic acid contents was found between the BG and the CG. However, bioaugmentation significantly improved the non-volatile acid, lactic acid, reducing sugar, and amino nitrogen contents, which are the important quality indices for Chinese cereal vinegar. The fermentation period of the BG was 5 days less than that of the CG. Starch utilization was also improved from 79% to 83% by bioaugmentation.

**TABLE 1:** Major indices of vinegar and evaluation of the effect of intensified fermentation.

	CG	BG	Improvement	*T*-test (*P*-value)
Total acid (g/100 ml)	3.56 ± 0.16	3.62 ± 0.21	<5%	0.714
Non-volatile acid (g/100 ml)	0.87 ± 0.24	1.33 ± 0.16	53%	0.049*
Lactic acid (g/100 ml)	1.59 ± 0.09	1.81 ± 0.02	14%	0.014*
Acetic acid (g/100 ml)	2.82 ± 0.03	2.84 ± 0.05	<5%	0.584
Amino nitrogen (g/100 ml)	0.17 ± 0.01	0.22 ± 0.02	30%	0.018*
Reducing sugar (g/100 ml)	0.82 ± 0.04	1.12 ± 0.02	37%	0.002**

** indicates there is a significant difference at P < 0.01 level, * indicates there is a significant difference at P < 0.05 level.

### Effect of Bioaugmentation on Bacterial Community

The bacterial communities were compared in this study. A total of 148 OTUs (relative abundance of more than 0.5%) was detected in the BG samples, four OTUs (two *Lactobacillus*, one *Nevskia*, and one *Pseudomonas*) more than those in the CG samples. However, the numbers of genera in the BG and CG were the same. This result indicated that bioaugmentation by *P. acidilactici* AAF1-5 could promote the growth of some other microorganisms.

**FIGURE 4:**
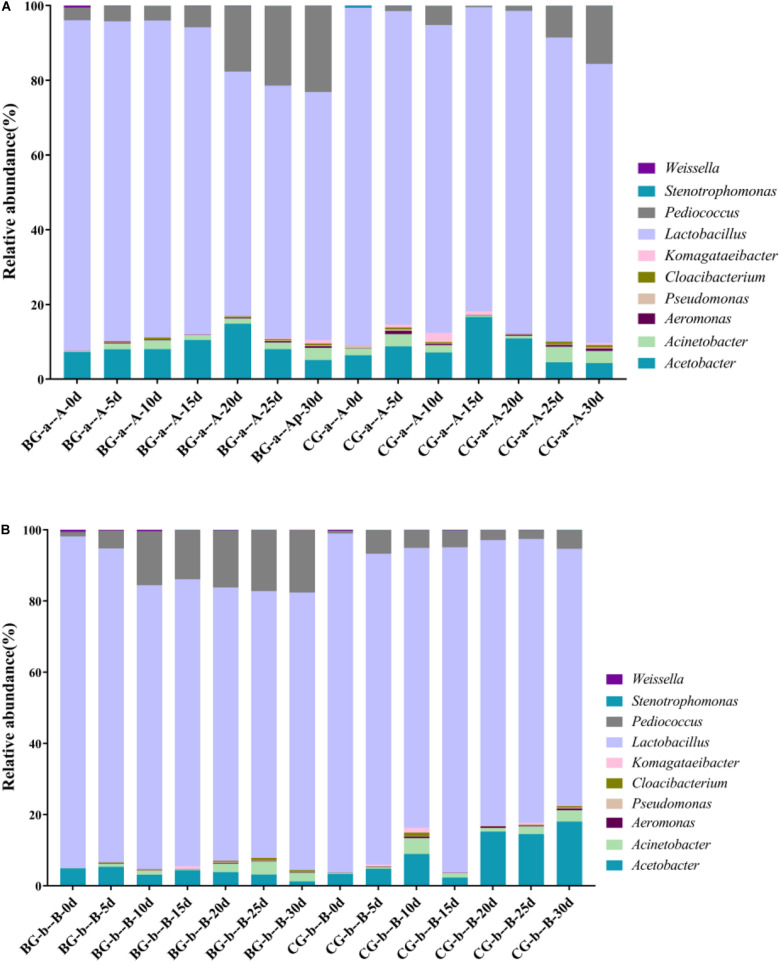
Bacterial community during fermentation. **(A)** *Cupei* “a–A,” **(B)** *Cupei* “b–B.”

The bacterial community successions in BG and CG fermentation were analyzed. The LAB and AAB genera in the BG were the same as those in the CG; however, their relative abundances differed (Figure 4). In *Cupei* “a–A,” *Lactobacillus*, *Pediococcus*, and *Acetobacter* were the predominant bacteria. The relative abundance of *Pediococcus* increased from 1.00 to 16.31% (30 d) in the *Cupei* “a–A” of the BG and increased from 1.02% to 5.91% in the CG. The abundance of *Lactobacillus* in the *Cupei* “a–A” of the BG was higher than that in the CG. Thus, the growth of other LAB and AAB was also improved due to the bioaugmentation of *P. acidilactici* AAF1-5. The increase in the functional microorganisms could promote the fermentation; thus, the production rate of total acid was improved by bioaugmentation (Figure 3). These differences in the microorganisms’ abundance could be the main cause of the different fermentation processes between the BG and the CG.

**FIGURE 5:**
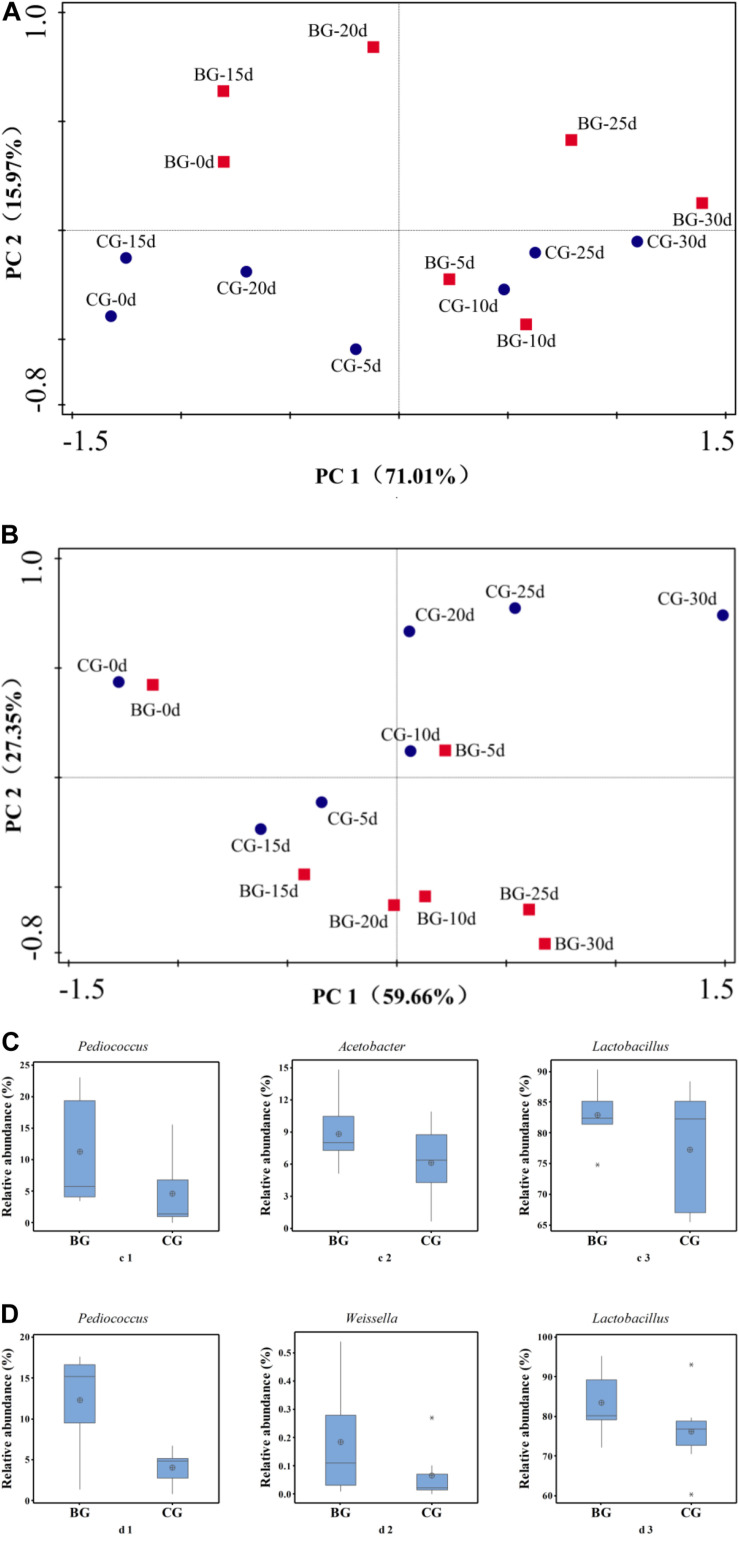
Principal component and metastats analyses of bacterial community. **(A)** PCA of “a–A” *Cupei*, **(B)** PCA of “b–B” *Cupei*, **(C)** metastats group difference analysis of *Cupei* “a–A,” **(D)** metastats group difference analysis of *Cupei* “b–B.”

PCA and Metastats group difference analyses were used to analyze the key genera that caused the difference in bacterial community. Based on PCA analysis, early stage I, middle stage I, early stage II, and middle stage II were the main different stages between the *Cupei* “a–A” of the two groups (Figure 5A). *Pediococcus*, *Acetobacter*, and *Lactobacillus* played a major role in the different bacterial community structures of *Cupei* “a–A” (Figure 5C), whose contents were higher in the BG than in the CG. In *Cupei* “b—B,” the difference appeared from middle stage I until the end of fermentation (Figure 5B). Even though no *P. acidilactici* AAF1-5 was added at the start of AAF, it spread in the fermenter due to the mass transfer from turning over *Cupei*. The microorganisms that caused the most difference was *Pediococcus*, followed by *Weissella* and *Lactobacillus* (Figure 5D). Therefore, not only the lactic acid production was improved, but also the acetic acid production, as described in Figures 3C,D, indicating that bioaugmenting *P. acidilactici* AAF1-5 could improve the growth and metabolism of these microorganisms.

## Discussion

Acetic acid and lactic acid are the main source of the acidic taste of Chinese traditional vinegars. Some non-volatile organic acids, mainly lactic acid, could reduce the sharpness of the flavor and taste of vinegar. Amino acid (amino nitrogen) and reducing sugar are important for the vinegar flavor. Their main source is the hydrolysis of macromolecular compounds, including protein, polysaccharides, and starch, by microorganisms (Li et al., 2016). In the present study, the relationship between the microorganisms and the physicochemical properties during AAF was highly correlated. *Acetobacter* and *Komagataeibacter* showed a significant positive correlation with total acid and acetic acid and a significant negative correlation with alcohol. In addition, non-volatile acid and lactic acid were highly correlated with the stage I samples and the microorganisms of *Lactobacillus* and *Pediococcus*, followed by *Weissella* and *Propionibacterium* (Figure 2D). These results could possibly serve as indication to adjust the fermentation, especially the acetic and lactic acid contents, through bioaugmentation.

As stress factors, total acid and temperature could affect the growth and metabolism of microorganisms, and a large number of microorganisms could disappear with the increase in acidity and temperature (Zheng et al., 2018). AAB, mainly *Acetobacter* and *Komagataeibacter*, have an excellent tolerance against the acids from vinegar, which are usually used in acetic acid fermentation. AAB accumulate acetic acid as a result of alcohol oxidization by membrane-bound pyrroloquinoline quinone-dependent alcohol dehydrogenase and aldehyde dehydrogenase (Qi et al., 2014). This bioconversion process is very quick. Thus, a small amount of AAB (7.43% of total bacteria) could oxidize the alcohol quickly (average of 0.68 g/100 g *Cupei*/d, Figure 2B). LAB mainly produces lactic acid by metabolizing sugars. In the AAF of TDMV, the LAB content increased slightly and then decreased. The average production rate of lactic acid was 0.21 g/100 g *Cupei*/d (Figure 2B), which was slower than the acetic acid production rate. These results indicated that LAB bioaugmentation could be a rational method to improve the utilization of carbohydrates in substrates and the flavor of vinegar.

Before bioaugmentation is applied, the special operation to turn over *Cupei* should be considered. In the AAF of TDMV, the oxygen content in the lower layer of *Cupei* was low because *Cupei* was not turned over and its depth limited the transfer from the air. Low oxygen content is favorable to the growth and metabolism of anaerobic and facultative aerobic microorganisms. Thus, lactic acid was mainly produced in the lower layer of *Cupei*, whereas acetic acid was mainly produced in the upper layer of *Cupei*. In *Cupei* “a,” rapid acetic acid oxidation resulted in high temperature and high acidity (Figure 3). Acid and thermal stresses were the preliminary challenge of strain, which was used for bioaugmentation. This research presented a rational protocol for bioaugmentation in solid-state fermentation of Chinese cereal vinegar. CCA was used to reveal the potential function of microorganisms and their relationship with fermentation conditions (Chen et al., 2020). A tolerant strain of *P. acidilactici* AAF1-5 was selected for bioaugmentation. Lactose was used as a thermal protectant for the microbial agent preparation of *P. acidilactici* AAF1-5. It is a kind of sugar contained in *Cupei* and does not affect the flavor of vinegar.

In addition, bioaugmentation could improve the acetification process and increase the bacterial diversity of TDMV. The BG exhibited four more OTUs than the CG. Moreover, the α-diversity of the bacterial community between the BG and the CG significantly differed (Table 2). The ACE, Chao1, and Shannon values of the BG were higher than those of the CG, whereas the Simpson index of the BG was lower than that of the CG. These results indicated that the bacterial community diversity of the BG was improved by the bioaugmentation of *P. acidilactici* AAF1-5. By comparing the microorganism community composition, the main different bacteria between BG and CG were *Lactobacillus* and *Pseudomonas*. The relative abundant of *Lactobacillus* were 82.25% and 74.79% on 15 day and 30 day at “a” and “A” *Cupei* of BG, respectively, which were 81.37% and 66.42% of CG. They were 88.51% and 77.97% on 15 day and 30 day at “b” and “B” *Cupei* of BG, and 84.76% and 72.13% of CG, respectively. The relative abundant of *Pseudomonas* were 0.06% and 0.23% on 15 day and 30 day at “a” and “A” *Cupei* of BG, and 0.03% and 0.21% of CG, respectively. They were 0.06% and 0.24% at “b” and “B” *Cupei* of BG, and 0.04% and 0.12% of CG. Thus, we supposed that the improvement of TDMV fermentation might be related with the enhancement of *Lactobacillus* sp. because it has been reported that *Lactobacillus* can produce amylase and protease, including *Lactobacillus amylophilus*, *Lactobacillus amylovorus*, *Lactobacillus plantarum* and *Lactobacillus fermentum* (Naveena et al., 2005; Rodriguez-Sanoja et al., 2005; Landete et al., 2014; Kanpiengjai et al., 2015; Kocabay et al., 2016). In our other research, metatranscriptomic approach was used to reveal the functions of microbiota in SSF of Chinese cereal vinegar, and the results showed that genera of *Lactobacillus* contributed a lot of expression of glucosidase, amylase, and protease and peptidase (data not shown). Moreover, lots of LAB are more thermal tolerant than acetic acid bacterial (Zhang et al., 2020). That might be the reason for higher bacterial community diversity of BG when compared with that of CG. LAB bioaugmentation was only performed in the upper layer of *Cupei*. In stage I, the bioaugmentation of *P. acidilactici* AAF1-5 improved the lactic acid production from carbohydrate due to its tolerance against acidity and temperature. In stage II, *P. acidilactici* AAF1-5, which spread into *Cupei* “b-B,” improved the lactic acid production and utilization of carbohydrates. The acetic and lactic acid production rates of the BG were higher than those of the CG. In *Cupei* “a” of the BG, the average production rates of acetic acid and lactic acid were 4.89% and 157.27% higher than those of the CG, respectively; in *Cupei* “b” of the BG, these rates were 1.29% and 71.88% higher than those in the CG, respectively. The temperatures of BG increased more quickly than that of CG due to the bioaugmentation (Figure 3). These results indicated that the metabolic activity of bacteria was improved by the bioaugmentation. Therefore, we speculated that at the early stage of fermentation bioaugmentation improved the metabolic activity, resulting to the quick increase of temperature and acidity. And then the bacterial community diversity increased due to the tolerance of some *Lactobacillus* sp. against thermal and acidity, which might improve the starch utilization by expressing enzymes to degrade starch. Moreover, the increase in the acidity also contributed to the decomposition of starch. Therefore, starch utilization was improved from 79% to 83% by bioaugmentation.

**TABLE 2:** Comparison of bacterial community diversity.

α-diversity	BC (*Cupei* ‘a*-*-A’)	BG (*Cupei* ‘a*-*-A’)	BC (*Cupei* ‘b*-*-B’)	BG (*Cupei* ‘b*-*-B’)
ACE	110.419 ± 4.388	113.767 ± 4.815*	99.572 ± 3.102	108.392 ± 3.453**
Chao1	111.206 ± 6.395	117.654 ± 4.733**	96.800 ± 5.853	111.963 ± 6.647*
Shannon	1.815 ± 0.244	1.921 ± 0.124**	1.821 ± 0.114	1.852 ± 0.177*
Simpson	0.533 ± 0.090	0.503 ± 0.095*	0.568 ± 0.066	0.538 ± 0.035*

** indicates there is a significant difference at P < 0.01 level, * indicates there is a significant difference at P < 0.05 level.

## Conclusion

Bioaugmentation technology was proved an effective strategy to improve the SSF of cereal vinegar. Before applying bioaugmentation, the rational analysis and designation are necessary. Fermentation process of TDMV was highly correlated with *Acetobacter* and *Lactobacillus*. Strain of *P. acidilactici* AAF1-5 could colonize and improve TDMV fermentation due to its acidification capacity and thermal tolerance. Bioaugmentation fermentation of TDMV by *P. acidilactici* AAF1-5 was five days less than that of the control, and the utilization of starch was improved from 79% to 83% by increasing the bacterial activity and the bacterial community diversity.

## Data Availability Statement

The datasets presented in this study can be found in online repositories. The names of the repository/repositories and accession number(s) can be found below: https://www.ncbi.nlm.nih.gov/genbank/, SRR11951261.

## Author Contributions

QZ, CZ, and YZ performed the experiments and substantially contributed to the acquisition, analysis, and interpretation of data. XW and XL were involved in the experiments. JS and MX were also involved in the experiments and revised and discussed the manuscript. RZ and MW designed the study and were involved in drafting and revising the manuscript. All authors have read and approved the manuscript.

## Conflict of Interest

RZ was employed by the company Tianjin Tianli Duliu Mature Vinegar Co., Ltd. The remaining authors declare that the research was conducted in the absence of any commercial or financial relationships that could be construed as a potential conflict of interest.

## Footnotes

1 https://www.ncbi.nlm.nih

2 http://www.ncbi.nlm.nih.gov/Traces/sra
