# Supplementary material for: Bioaugmentation by Pediococcus acidilactici AAF1-5 Improves the Bacterial Activity and Diversity of Cereal Vinegar Under Solid-State Fermentation
Source: Front Microbiol. 2021 Jan 28;11:603721. doi: 10.3389/fmicb.2020.603721 (PMC7876233; doi:10.3389/fmicb.2020.603721)
Supplement: Supplementary file 1 [file Table_1.DOCX]

**Applied Microbiology and Biotechnology**

**Bioaugmentation improves the bacterial activity and diversity of cereal vinegar under solid–state fermentation**

Zhang Qiang^1^, Zhao Cuimei^1^, Fu chuanxue^1^, Zheng Yu^1*^, Jia Song^1^, Wang xiaobin^1^, Li xiaowei^1^, Xia Menglei^1^, Zhang Rongzhan^2^, Wang Min^1*^

1. State Key Laboratory of Food Nutrition and Safety. Key Laboratory of Industrial Fermentation Microbiology, Ministry of Education. College of Biotechnology, Tianjin University of Science and Technology, Tianjin 300457, China.

2.Tianjin Tianli Duliu Mature Vinegar CO., LTD., Tianjin 301602, China.

* Corresponding Author

Tel: +86-22-60601256. Fax: +86-22-60602298.

E-mail: [yuzheng@tust.edu.cn](mailto:yuzheng@tust.edu.cn)











Fig. 1 Fermentation and tolerance characteristics of different lactic acid bacteria

(A) Acid production characteristics; (B) thermal tolerance; (C)acidity tolerance
